# Effectiveness of price-reduced meals on purchases among university young adults

**DOI:** 10.1017/jns.2021.87

**Published:** 2021-10-29

**Authors:** Rajshri Roy, Kate Harrington

**Affiliations:** Discipline of Nutrition and Dietetics, The University of Auckland, School of Medical Sciences, Auckland, New Zealand

**Keywords:** Food choice, Healthy eating, Nutrition intervention, Price, Young adults, POP, point-of-purchase

## Abstract

University food environments influence dietary behaviours of attending young adults (aged 18–35 years). The present study aimed to determine if price-reduced meals are associated with high purchase volumes at food outlets (*n* 5) in a large urban university. The university food outlet customers: university staff and students (*n* 244) were surveyed about their food choice determinants and their awareness of a price-reduced meal initiative called ‘Budgie Meals’. Itemised sales of ‘Budgie Meals’ and other meals across 3 years were collected. The ‘Budgie Meals’ were nutritionally analyzed. The *χ*^2^ and Mann–Whitney *U* tests were used to analyze quantitative survey responses. An open-ended item was thematically analyzed. Itemised sales of ‘Budgie Meals’ were measured across 3 years and were analyzed using the analysis of variance. The ‘Budgie Meals’ were nutritionally analyzed and categorised as ‘green,’ ‘amber’ or ‘red’ using the National Healthy Food and Drink Policy. Price was considered the most significant barrier to healthy food purchases. The awareness of the ‘Budgie Meal’ initiative was poor. The ‘Budgie Meal’ had higher sales volumes at each outlet than other items, but the sales showed a downward trend across the years. Nutritional analyses revealed that ‘Budgie Meals’ could be improved. The researchers suggested nutritional improvements to food retailers. Further research is required to assess the viability of implementing such nutritional improvements across food outlets. Specifically, collaboration with retailers and customers is needed to establish the economic feasibility, any potential revenue losses and testing taste acceptability of recipe alterations to these price-reduced meals.

## Introduction

The ‘food environment’ refers to the number, type and accessibility of food outlets, and the availability, cost, quality and promotion of food and beverage products^(1)^. Young adults (aged 18–35 years) may be particularly vulnerable to the food environment's features, such as food cost, as they typically have lower disposable incomes^(2)^. Young adults have a higher intake of energy-dense, nutrient-poor foods and drinks, including sugar-sweetened soft drinks, fried potatoes, meat pies, savoury pastries, pizza, crisps and confectionery compared with older adults^(3,4)^. The years at college or university is a period characterised by changes in eating behaviour in young adults^(5–9)^. Evidence indicates that young adults’ average weight gain in their first year at university is approximately 3⋅38 kg^(3)^. A large proportion of young adults attend tertiary education institutions such as universities. In 2019/20, there were estimated to be over 2⋅53 million students enrolled in tertiary education institutions in the UK with 40 % of students aged between 19 and 24 years^(10)^. There were approximately 19⋅6 million college students in the USA in 2019^(11)^. In New Zealand, the study setting, there were 175 240 university students in 2018^(12)^. The high attendance in tertiary institutions and the often-closed nature of these environments in the provision of foods and beverages to young adults, including students and staff, have the substantial potential to influence dietary behaviours in this population^(7)^.

Individual food choices are influenced by a wide variety of environmental and individual variables^(2)^. Three main dimensions of food choices are taste, perceived value (including price and portion size) and perceived nutrition^(2,13–17)^. Recent studies have applied economic theories to changing dietary behaviours^(13)^. One strategy to help young adults make healthier food choices comprises price manipulations. Price reduction strategies promote targeted foods by lowering their cost relative to alternative food choices^(13,18–22)^. Although food price is shown to be an important determinant of young adults’ eating behaviour, there is limited evidence of this being investigated in a real-world setting^(23)^. Instead, pricing strategies have been limited to experimental models in the tertiary environment to show the effect of a reduced price on purchasing healthy foods^(13)^. This cross-sectional observational study aimed to observe the food sales of price-reduced meals on menus (called ‘Budgie Meals’) in a real-world tertiary education setting. Using surveys at point-of-purchase (POP), the present study aimed to gain further insight into the reasons why price-reduced meals did or did not change purchasing behaviours.

The present study aimed to address the following research questions:

1. What are the key determinants influencing the food purchases of young adults attending university?
2. Are university young adults aware of price-reduced meal initiative, and does this awareness influence their purchasing?
3. Do price-reduced meals sell more than other menu items at five food outlets at a large urban university?
4. How can price-reduced meals be used as an avenue to increase the purchase of healthful foods in young adults attending university?

## Methods

### Study design

A cross-sectional observational study was conducted within a large, urban university campus with over 40 000 students and over 4000 staff. Five food outlets that offered a price-reduced meal option on campus and could provide itemised food sales data were selected for the present study. The five outlets together on average serve 400 meals per weekday. Price-reduced meals were compared with other meals regarding their sales and nutritional values. Customers were asked about their food purchase behaviours.

The present study was conducted according to the guidelines laid down in the Declaration of Helsinki, and all procedures involving human subjects/patients were approved by the University of Auckland Human Research Ethics Committee on 7 November 2018 for 3 years (Reference No. 022075). Written informed consent was obtained from all customers.

#### Price-reduced meals

**Fig. 1.**
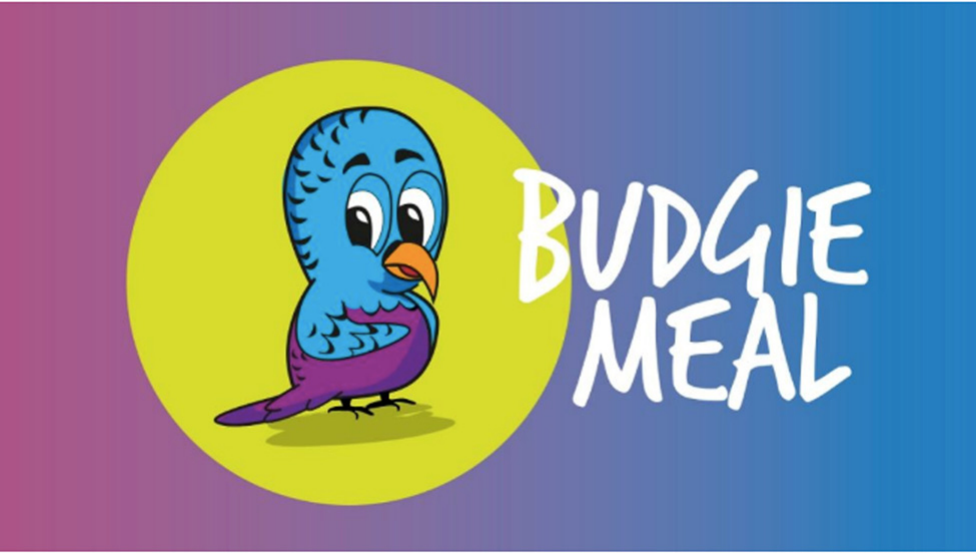
Price-reduced ‘Budgie Meal’ symbol.

The ‘Budgie Meal’ is a price-reduced meal option available at most food outlets, offering staff and students the opportunity to purchase a substantial meal from an array of cuisines across campus. The meals are $6⋅50 or under and consist of protein, vegetables and carbohydrates. The budgie meals are promoted at the outlets with a symbol (Fig. 1) or described as the ‘Meal of the Day’.

#### POP intercept survey

POP intercept surveys were conducted in convenient subsamples of customers to assess influences on food choice. A researcher intercepted customers by approaching individuals at each food outlet and inviting them to complete a paper-based survey after placing an order for a menu item. Customers needed to have purchased an item at the food outlet and not previously completed the survey to be eligible for participation. The researcher explained the present study's purpose, their involvement, the monetary incentive (draw to win a $20 supermarket voucher) and an opportunity to read the participant information sheet.

The survey was designed to collect data to investigate the key determinants of food purchases in these customers, the awareness of the ‘Budgie Meal’ initiative on campus and the influence of this price-reduced initiative on purchases. The survey questionnaire and the intercept methodology have been previously pilot-tested by the researchers^(24)^. The initial survey items collected demographic information from participants. Participants were then asked to report what they had purchased in a free text box. A survey question included a rating scale that asked participants to rank a purchasing factor on a scale of 1–5 (with 1 being most important and 5 being least important). The purchasing factors included taste, preference (the evaluative attitudes people express towards food)^(25)^, price, convenience and health/nutrition. Closed-ended survey items then prompted participants to report whether they were aware of the ‘Budgie Meal’ price-reduced meal initiative. If participants selected ‘yes’ to noticing the ‘Budgie Meal’ initiative on the menu, they were then asked if it influenced their purchase and to report what they purchased. Two closed-ended questions then asked participants to report if they thought having a ‘cheaper option’ and a ‘cheaper option which is also the healthier choice’ would influence their future purchases. A multiple-choice question asked participants to state the average number of times they purchase from university food outlets. Participants were then asked to select two significant barriers to purchasing food on campus. The purchasing barriers included a limited selection of healthy options, unaware of available healthy options, price, taste, preference and/or other (specify). The final open-ended survey item asked participants to report any improvements they wanted to see for the ‘Budgie Meals’ initiative.

### Data collection

#### Survey

Intercept survey data were collected over two university semesters in 2019. Survey data collection was conducted at the five food outlets involved in the present study. The survey took approximately 3–5 min for participants to complete. This was done while participants were waiting for their items to be ready. During popular (heavily-trafficked) periods, survey data collection took place, such as lunchtime (between 11:30 and 13:45 ), to ensure that the maximum number of participants was intercepted each time. One or two researchers were sent out to recruit as many customers as possible across all five outlets. The timeframe of data collection was informed by discussions with the retail manager of each food outlet.

#### Food sales

Itemised food sales data were collected from the five food outlets across 3 years (2017, 2018 and 2019) except for one outlet, which was not operating in 2017. Data included the name of the top 5 performing menu items, the name of the ‘Budgie Meal’ items on offer and the corresponding number of each item sold at each food outlet. 3 years’ worth of data were collected to observe trends in the ‘Budgie Meal’ sales over time. Food sales data were collected in electronic receipts provided by the retail manager of each food outlet.

#### Nutritional analyses of ‘Budgie Meals’

The researchers collected detailed recipes for the top five performing menu items and ‘Budgie Meal’ items from the retail managers. The menu items from the food outlet were analysed by a registered dietitian from the standard recipes supplied by the food retailers using FoodWorks software^(26)^ that uses the New Zealand database of foods. These menu items were then categorised into healthy (green), intermediate (amber) and unhealthy (red) using established criteria within the National Food and Drink Policy implemented in New Zealand's hospital foodservice outlets^(27)^.

### Statistical analysis

Data were analyzed using IBM SPSS Statistics software (version 25), GraphPad Prism software for Mac (version 8.2.0), FoodWorks^(26)^ (version 10, New Zealand database) and Microsoft Excel for Mac (version 16.23). Significance was determined by a *P*-value of <0⋅05 for all statistical analyses. Descriptive statistics were performed to establish the proportions, percentages, mean and standard deviations of the participants’ demographic information. Percentages were also calculated to determine participant survey responses for each survey question. Statistical analyses of the quantitative survey data were conducted using the *χ*^2^ test for association and the Mann–Whitney *U* test. Inductive thematic analysis of the open-ended survey question was conducted on Microsoft Excel. The one-way analysis of variance (ANOVA) was used to determine any statistically significant differences between the ‘Budgie Meal’ sales each year and between each outlet. A Bonferroni *post hoc* test was conducted to identify which of the specific groups differed. The top 5 performing menu items and ‘Budgie Meal’ items for each food outlet were nutritionally analyzed using the New Zealand database of FoodWorks 10 software. These were then categorised into ‘green,’ ‘amber’ and ‘red’ foods based on the National Food and Drink Policy.

## Results

### Survey results

#### Demographic characteristics of respondents

Two hundred and eighty-eight customers completed the survey; forty-three incomplete surveys were excluded. The survey participants were staff and students (aged 18–35 years) of the university purchasing at the food outlet. Table 1 lists the characteristics of those who completed the survey (*n* 244).

**Table 1.** Participant demographic characteristics

Characteristic	*N*	Percentage (%)
Total participants	244	–
Gender		
Male	89	36⋅5
Female	155	63⋅5
Age		
Mean age	22⋅0	–
Standard deviation	3⋅8	–
18–24	196	80⋅3
25–35	48	19⋅7
Occupation		
Staff	29	11⋅9
Student	209	85⋅7
Both (staff and student)	5	2⋅0
Other	1	0⋅4

#### Awareness and influence of the price-reduced meals on purchases

A total of thirteen participants (5 %) reported purchasing a price-reduced ‘Budgie Meal’, while 206 participants (85 %) reported not purchasing one. A further twenty-five participant (10 %) did not know whether they had purchased a ‘Budgie Meal’. Most participants (72⋅5 %) did not notice the ‘Budgie Meal’ symbol for the price-reduced meal, while 27⋅5 % did notice it. The symbol influenced the purchases of 4⋅5 % of participants. There were no significant differences between gender, age and occupation groups (Table 2).

**Table 2.** Survey responses to questions on the awareness, influence and perceived influence in the future of the ‘Budgie Meal’ initiative, and price as a barrier to purchasing food on the university campus

Demographic	Yes	No	*P*-value
	*N* (%)	*N* (%)	
Did you notice the Budgie Meal symbol today?			
Participants	67 (27⋅5)	177 (72⋅5)	
Gender			
Male	23 (25⋅8)	66 (74⋅2)	0⋅917
Female	41 (26⋅5)	114 (73⋅5)	
Age range			
18–24	47 (24)	149 (76)	0⋅106
25–35	17 (35⋅4)	31 (64⋅6)	
Occupation			
Staff	11 (37⋅9)	18 (62⋅1)	0⋅120
Student	51 (24⋅4)	158 (75⋅6)	
Did the Budgie Meal symbol influence your purchase today?			
Participants	11 (4⋅5)	91 (37⋅3)	
Gender			
Male	6 (15⋅4)	33 (84⋅6)	0⋅356
Female	6 (9⋅4)	58 (90⋅6)	
Age range			
18–24	9 (10⋅7)	75 (89⋅3)	0⋅533
25–35	3 (15⋅8)	16 (84⋅2)	
Occupation			
Staff	2 (16⋅7)	10 (83⋅3)	0⋅504
Student	9 (10⋅2)	79 (89⋅8)	
Is price a barrier to purchasing healthy food options?			
Participants	182 (74⋅5)	62 (25⋅4)	
Gender			
Male	59 (66⋅3)	30 (33⋅7)	0⋅024*
Female	123 (79⋅4)	32 (20⋅6)	
Age range			
18–24	156 (79⋅6)	40 (20⋅4)	0⋅000*
25–35	26 (54⋅2)	22 (45⋅8)	
Occupation			
Staff	15 (51⋅7)	14 (48⋅3)	0⋅003*
Student	162 (77⋅5)	47 (22⋅5)	
Do you believe that having a cheaper option would influence your choice in the future?			
Participants	214 (87⋅7)	30 (12⋅3)	
Gender			
Male	78 (87⋅6)	11 (12⋅4)	0⋅981
Female	136 (87⋅7)	19 (12⋅3)	
Age range			
18–24	177 (90⋅3)	19 (9⋅7)	0⋅012*
25–35	37 (77⋅1)	11 (22⋅9)	
Occupation			
Staff	23 (79⋅3)	6 (20⋅7)	0⋅111
Student	187 (89⋅5)	22 (10⋅5)	
Do you believe that having a cheaper option which is also the healthier choice would influence your choice in the future?			
Participants	213 (87⋅3)	31 (12⋅7)	
Gender			
Male	72 (80⋅9)	17 (19⋅1)	0⋅023*
Female	141 (91)	14 (9)	
Age range			
18–24	173 (88⋅3)	23 (11⋅7)	0⋅358
25–35	40 (83⋅3)	8 (16⋅7)	
Occupation			
Staff	25 (86⋅2)	4 (13⋅8)	0⋅896
Student	182 (87⋅1)	27 (12⋅9)	

* *P*-values below 0⋅05 were considered statistically significant.

#### Food purchasing behaviours and determinants

Taste was reported as the most important factor influencing most participants’ (36⋅5 %) purchases. Price was the most important factor chosen by 15⋅6 % of participants. Health/nutrition was ranked lowest as an influencing factor by 38⋅5 % of the participants. There was a significant difference in the ranking (1–5) of health/nutrition between the different age groups (18–24- and 25–35-year-olds). In the 18–24 age group, 5⋅6 % gave health/nutrition a ranking of 1 compared with the 25–35 age group, where 14⋅6 % gave health/nutrition a ranking of 1 (*P* < 0⋅001). A further 43⋅4 % of the 18–24-year-olds ranked health/nutrition as 5 (least important), whereas only 25 % of the 25–35-year-olds gave this factor a ranking of 5. Of the respondents who reported being influenced by the ‘Budgie Meal’ initiative, 63⋅6 % ranked price at 1, whereas 16⋅5 % of participants who reported not being influenced by the ‘Budgie Meal’ initiative ranked price as 1 (*P* < 0⋅001). Amongst those who reported purchasing a ‘Budgie Meal’, 36⋅4 % of them ranked price as 1 (most important), and none of them ranked price as 5 (least important) (*P* < 0⋅005). In contrast, amongst participants who reported not purchasing a ‘Budgie Meal’, 19⋅3 % of these participants ranked price as 5 (least important) (*P* < 0⋅005) (Table 3).

**Table 3.** Survey responses to factors influencing purchases of food at the university between different participant groups

Age	18–24	25–35	
Ranking for each factor	*N* (%)	*N* (%)	*P*-value
Health/nutrition			
1	11 (5⋅6)	7 (14⋅6)	0⋅000*
2	21 (10⋅7)	13 (27⋅1)	
3	32 (16⋅3)	11 (22⋅9)	
4	50 (25⋅5)	5 (10⋅4)	
5	82 (43⋅4)	12 (25)	
Preference			
1	25 (12⋅8)	5 (10⋅4)	0⋅043*
2	41 (20⋅9)	6 (12⋅5)	
3	50 (25⋅5)	10 (20⋅8)	
4	38 (19⋅4)	10 (20⋅8)	
5	42 (21⋅4)	17 (35⋅4)	
Price			
1	33 (16⋅8)	5 (10⋅4)	0⋅221
2	42 (21⋅4)	9 (18⋅8)	
3	43 (21⋅9)	11 (22⋅0)	
4	43 (21⋅9)	13 (27⋅1)	
5	35 (17⋅9)	10 (20⋅8)	
Taste			
1	70 (35⋅7)	19 (39⋅6)	0⋅934
2	57 (29⋅1)	11 (22⋅9)	
3	43 (21⋅9)	9 (18⋅8)	
4	16 (8⋅2)	5 (10⋅4)	
5	10 (5⋅1)	4 (8⋅3)	
Convenience			
1	57 (29⋅1)	12 (25)	0⋅742
2	35 (17⋅9)	9 (18⋅8)	
3	28 (14⋅3)	7 (14⋅6)	
4	50 (25⋅5)	15 (31⋅3)	
5	26 (13⋅3)	5 (10⋅4)	
Influenced by the ‘Budgie Meal’ initiative			
1	1 (9⋅1)	8 (8⋅8)	0⋅265
2	1 (9⋅1)	15 (16⋅5)	
3	3 (27⋅3)	18 (19⋅8)	
4	0 (0)	21 (23⋅1)	
5	7 (63⋅6)	29 (31⋅9)	
Preference			
1	0 (0)	13 (14⋅3)	0⋅226
2	2 (18⋅2)	18 (19⋅8)	
3	3 (27⋅3)	24 (26⋅4)	
4	4 (36⋅4)	14 (15⋅4)	
5	3 (27⋅3)	22 (24⋅2)	
Price			
1	7 (63⋅6)	15 (16⋅5)	0⋅003*
2	1 (9⋅1)	18 (19⋅8)	
3	3 (27⋅3)	16 (17⋅6)	
4	1 (9⋅1)	22 (24⋅2)	
5	0 (0)	20 (22)	
Taste			
1	4 (36⋅4)	34 (37⋅4)	0⋅574
2	3 (27⋅3)	25 (27⋅5)	
3	1 (9⋅1)	17 (18⋅7)	
4	3 (27⋅3)	6 (6⋅6)	
5	1 (9⋅1)	9 (9⋅9)	
Convenience			
1	0 (0)	21 (23⋅1)	0⋅720
2	5 (45⋅5)	15 (16⋅5)	
3	2 (18⋅2)	16 (17⋅6)	
4	4 (36⋅4)	28 (30⋅8)	
5	1 (9⋅1)	11 (12⋅1)	
Purchased a ‘Budgie Meal’			
1	1(9⋅1)	17 (7⋅3)	0⋅245
2	2 (18⋅2)	32 (13⋅7)	
3	4 (36⋅4)	39 (16⋅7)	
4	1 (9⋅1)	54 (23⋅2)	
5	3 (27⋅3)	91 (39⋅1)	
Preference			
1	0 (0)	30 (12⋅9)	0⋅004*
2	0 (0)	47 (20⋅2)	
3	3 (27⋅3)	57 (24⋅5)	
4	1 (9⋅1)	47 (20⋅2)	
5	7 (63⋅6)	52 (22⋅3)	
Price			
1	4 (36⋅4)	34 (14⋅6)	0⋅025*
2	3 (27⋅3)	48 (20⋅6)	
3	2 (18⋅2)	52 (22⋅3)	
4	2 (18⋅2)	54 (23⋅2)	
5	0 (0)	45 (19⋅3)	
Taste			
1	4 (36⋅4)	85 (36⋅5)	0⋅820
2	4 (36⋅4)	64 (27⋅5)	
3	1 (9⋅1)	51 (21⋅9)	
4	2 (18⋅2)	19 (8⋅2)	
5	0 (0)	14 (6)	
Convenience			
1	2 (18⋅2)	67 (28⋅8)	0⋅444
2	2 (18⋅2)	42 (18)	
3	1 (9⋅1)	34 (14⋅6)	
4	5 (45⋅5)	60 (25⋅8)	
5	1 (9⋅1)	30 (12⋅9)	

* *P*-values below 0⋅05 were considered statistically significant.

#### Barriers to purchasing food at the university

Most respondents (74⋅6 %) selected price as their food purchasing barrier followed by a limited selection of healthy options (62⋅3 %) (Table 2), significantly more than taste (22⋅1 %), preference (13⋅9 %) and unaware of healthy options (8 %) (*P* < 0⋅001). There was a significant difference between the selection of price as a barrier between genders, as 79⋅4 % of females selected price over 66⋅3 % of males (*P* < 0⋅05). A significant difference was observed in price barriers between age groups, 79⋅6 % of 18–24-year-olds and 54⋅2 % of 25–35-year-olds (*P* < 0⋅001) selected price. Furthermore, a larger proportion of students (77⋅5 %) perceived the price as a barrier to purchasing healthy options, over 51⋅7 % of staff participants (*P* < 0⋅001) (Table 3).

#### Perceived influence of a healthier price-reduced meal in the future

Overall, 87⋅7 % of participants reported that a cheaper option would influence them in the future, while 12⋅3 % of participants did not believe it would affect their future purchases. There was a statistically significant difference between age groups, as 90⋅3 % of 18–24-year-olds reported that a price-reduced meal option would influence their purchase in the future compared with 77⋅1 % of 25–35-year-olds (*P* < 0⋅005). Most participants (87⋅3 %) reported that the availability of a healthier price-reduced meal option would influence their purchase in the future, with only 12⋅7 % reporting that it would not (Table 2).

#### Suggested improvements to price-reduced meals

The open-ended question asking for suggestions for improvements to the price-reduced meals was analysed using an inductive content analysis approach. The responses summarised into three themes: ‘adding more vegetables to the ‘Budgie Meals’, ‘offering ‘Budgie Meals’ at more food outlets’ and ‘increasing the portion size of the ‘Budgie Meals’.

### Food sales results

#### Food sales of ‘Budgie Meals’ compared with other top-selling items

Table 4 shows the itemised sales of the ‘Budgie Meals’ at each food outlet across 3 years. The ‘Budgie Meal’ was ranked amongst the most popular (top 5 selling) items at every food outlet in 2017 except outlet B, which did not offer a price-reduced meal. The ‘Budgie Meal’ was also ranked in the top 5 for all five outlets in 2018, except for one ranked sixth. However, in 2019, the ‘Budgie Meal’ only ranked inside the top 5 at two of the five food outlets. The declining trend of ‘Budgie Meal’ sales over 3 years was observed at each food outlet, except outlet D, where the price-reduced meal sales remained steady. Overall, there was no statistically significant difference found in the total ‘Budgie Meal’ sales across the five outlets between each year. However, there was a statistically significant difference in mean ‘Budgie Meal’ sales between the food outlets across the 3 years as determined by the one-way ANOVA (*F*(2,14) = 0⋅929, *P* = 0⋅028). A Bonferroni *post hoc* test revealed that the ‘Budgie Meal’ sales were significantly higher at outlet A than outlet B (*P* < 0⋅05).

**Table 4.** Number of ‘budgie meals’ sold as a percentage of total at each food outlet across the 3-year observation period

Year	Item	Outlet A	Outlet B	Outlet C	Outlet D	Outlet E
2017	No. of Budgie Meals sold	2099 (15 %)	No Budgie Meals available	442 (36 %)	912 (5⋅2 %)	1368 (19 %)
2018	No. of Budgie Meals sold	2285 (14⋅7 %)	479 (32 %)	581 (22 %)	1566 (5 %)	811 (7 %)
2019	No. of Budgie Meals sold	605 (9⋅4 %)	60 (5⋅7 %)	120 (13 %)	677 (4⋅5 %)	1289 (17⋅5 %)
2017–2019	Mean sales	1663⋅0	179⋅7	381⋅0	1051⋅7	1156⋅0
2017–2019	SD	921	261	236⋅5	460⋅7	301⋅4

There was a statistically significant difference in the mean number of ‘budgie meals’ sold between the outlets across the 3-year observation period as determined by the one-way ANOVA (*F*(2,14) = 0⋅929, *P* = 0⋅028). A Bonferroni *post hoc* test revealed that the ‘Budgie Meal’ sales were significantly higher at outlet A than outlet B (*P* < 0⋅05).

### Nutritional analyses of ‘Budgie Meals’

There was a larger proportion of top 5 performing menu items categorised as red (35 %) and green (22 %) items across all food outlets than the ‘Budgie Meals’. Specifically, 6 % of the ‘Budgie Meals’ on offer at the food outlets belonged in the green category, and 71 % belonged in the nutrient criteria’ amber category. Each food outlet had at least 50 % of their ‘Budgie Meal’ items belonging to the amber category. Outlet B had the largest proportion of ‘Budgie Meal’ items belonging to the amber category (100 %). Outlet D was the only food outlet to have ‘Budgie Meal’ items in the green category of the nutrient criteria (50 % of their ‘Budgie Meals’). Three out of five food outlets (outlet C, outlet A and outlet E) had ‘Budgie Meal’ items belonging to the red category. Using the nutritional analysis, the researchers identified ways to improve any ‘Budgie Meals’ classified as ‘amber’ or ‘red’ in the nutrient criteria to facilitate the progression of items into ‘green’ or ‘amber’ categories. Table 5 displays the suggested changes by a registered dietitian to these ‘Budgie Meals’.

**Table 5.** Suggested changes to improve the nutritional value of ‘Budgie Meals’ on offer at each food outlet

Outlet	‘Budgie Meal’	Classification	Recommendation to improve the nutritional value of the ‘Budgie Meal’
A	Bacon and mushroom carbonara	Red	Addition of vegetables, e.g. broccoli, peas and alternative pasta (wholemeal pasta).
	Blue cheese fettucine	Red	Reduction in the cream used or low-fat milk substitute to be used.
	Lamb tagine	Amber	Substitution of white rice to brown rice to increase wholegrains and fibre.
	Mushroom stroganoff	Amber	Addition of more vegetables and substitution of wheat pasta to wholemeal pasta.
	Puttanesca	Amber	Addition of protein, e.g. legumes.
	Ratatouille	Amber	Addition of protein, e.g. legumes and nuts. Substitution of white rice for brown rice.
	Spinach dhal	Amber	Substitution of white rice to brown rice to increase wholegrains and fibre.
	Vegetarian tagine	Amber	Substitution of white rice to brown rice to increase wholegrains and fibre.
B	Mushroom and carrot risotto	Amber	Alternative milk products to be used that are lower in energy and saturated fat, e.g. trim milk.
	Soup of the day	Amber	Alternative products to be used instead of the cream that is lower in energy and saturated fat, e.g. Greek yoghurt.
	Macaroni pasta	Amber	Alternative milk products to be used that are lower in energy and saturated fat, e.g. trim milk.
	Chickpea and potato curry	Amber	Substitution of white rice for brown rice.
	Vegetarian fried rice	Amber	Reduction in sodium and substitution of white rice for brown rice.
C	Mac and cheese	Amber	Reduced-fat dairy products used (cheese and milk). Wholemeal pasta to be used instead of wheat pasta.
	Chicken veggie mix with toast	Amber	Substitution of the white/plain bread for a wholegrain version.
	Creamy mushrooms and hash brown and toast	Red	Add more vegetables to the dish, e.g. spinach or roasted tomato on the side. Baked potato instead of a hash brown as an option.
	Potato and green pea curry with deep-fried bread	Amber	Have an option of carbohydrate that is lower in energy than the deep-fried bread, such as wholemeal roti. Add another vegetable into the curry, e.g. carrots.
	Potato and sausage bake	Red	Addition of vegetables to the dish, e.g. sweetcorn and cauliflower. Substitution of sausage for a less-processed option, e.g. mince.
D	Protein bowl	Green	No changes.
	Granola bowl	Green	No changes.
	Berry bowl	Amber	Addition of a protein source, e.g. milk, yoghurt and increased serving size.
	Tropical bowl	Amber	Increased serving size. Substitution of white rice for brown rice or another wholegrain such as quinoa or millet.
E	Creamy mushroom pasta	Red	Addition of vegetables (incorporated into the dish). Reduction in the cream used or replaced with lower-fat milk.
	Creamy spinach pasta	Red	Addition of vegetables (incorporated into the dish). Reduction in the cream used or replaced with lower-fat milk.
	Thai red curry	Amber	Substitution of white rice for brown rice.
	Thai green curry	Amber	Substitution of white rice for brown rice.
	Kimchi fried rice	Amber	Substitution of white rice for brown rice. Reduction in oil used.
	Mix veggie stew	Green	No changes.
	Ratatouille pasta	Amber	Wholegrain to be used instead of refined carbohydrate.
	Spicy pork on rice	Red	Substitution of white rice for brown rice. Addition of vegetables to the dish.

## Discussion

Study findings indicated that taste was the largest food purchasing determinant. Simultaneously, the price was the most significant barrier to healthy food purchases, followed by a limited selection of healthy options among young adults at a large urban university. Food sales revealed that the ‘Budgie Meals’ were a top-selling item at each food outlet. However, in the present study, the awareness and influence of the ‘Budgie Meal’ initiative at the POP were poor. The sales showed a downward trend across the years, and nutrient analyses indicated that the ‘Budgie Meals’ were of low nutritional quality.

The results are consistent with Tam *et al.* and Roy *et al.*'s research on the investigation into food purchasing determinants and the availability of healthy food in university food environments, where taste, price and a lack of healthy options influenced customers’ food purchases^(6,8)^. The experimental studies conducted by French *et al.* showed the positive effect of price interventions on sales volumes of healthier snack items^(28,29)^. Previous studies have highlighted that cheaper foods, such as price-reduced menu items, were generally less healthy in public settings such as universities^(7,8,30)^.

Under one-third of survey respondents noticed the ‘Budgie Meal’ price-reduced meal initiative at the POP. Survey results showed that the ‘Budgie Meal’ price-reduced meal initiative influenced only a small number of respondent purchases at the POP. A recent audit of the same university food environment indicated minimal promotion of food and beverage items at the POP across all outlets, regardless of nutritional value^(8)^. The researchers noticed that only a few outlets (outlet D, outlet E and outlet C) had a poster with the ‘Budgie Meal’ symbol and name advertised at the till. On the other hand, outlet B did not advertise for the ‘Budgie Meal’ option visible to consumers. Moreover, outlet A advertised their ‘Budgie Meal’ as ‘Meal of the Day’, leading to participants’ potential lack of association between the $6⋅50 meals and ‘Budgie Meals’. Thus, inconsistent advertising and visible promotion of the ‘Budgie Meal’ symbol at the POP may contribute to poor awareness amongst university staff and students. The declining sales trend across most outlets could be attributed to unawareness of the ‘Budgie Meal’ initiative and subsequent poor influence of the price-reduced initiative on purchases^(31,32)^. Promotion and education have been shown to have a significant independent effect on the percentage of snack sales^(13,33)^. Once the price-reduced meals have been nutritionally improved, evidence shows that increased promotion of healthy foods at the POP is associated with increased purchasing of these items^(13,28,29,32)^. This would have the effect of raising awareness of the initiative and potentially allowing the ‘Budgie Meal’ price-reduced initiative to influence university staff and student's purchases in the process^(34)^.

The price-reduced initiative was still attractive for individuals who considered the price to be of great importance when making their food choice. These findings are consistent with previous studies that found young adults were attracted to cheaper food items that provide ‘value for money’ due to this age group having less disposable income for food expenditure^(8,24,35)^. Moreover, Roy *et al.* identified ‘value for money’ as the second-largest food purchasing determinant in the same university population as the present study^(8,24)^. The present and other studies in the literature have found that health/nutrition ranks low in importance for young adults than other factors, including taste, value, accessibility and low cost. Emerging adulthood (18–24 years) is characterised by developing autonomous food choices, and managing a healthy diet is a low priority^(3)^. There was no statistically significant difference in the ranking of health/nutrition between genders. This is contrary to previous studies that found females placed greater importance on nutritional value and tended to purchase healthier options, while males tended to buy energy-dense foods on the go^(36,37)^. In the present study, both males and females do not consider health/nutrition at the POP. However, females may be more likely to be influenced by a cheaper and healthier option in the future if it was on the market. Participants in the present study wanted improvements to the ‘Budgie Meals’. The largest theme that emerged was ‘offering “Budgie Meals” at more food outlets’, followed by ‘adding more vegetables to the “Budgie Meals”’^(37)^.

Recipe analyses indicated that the ‘Budgie Meals’ were predominantly unhealthy. They had less nutritional value than other top-selling items at the food outlets. Improving the nutritional value of cheap ‘Budgie Meal’ items is vital in addressing cost barriers and lack of healthy options available for young adults in university settings^(32)^. Research suggests that healthy, fresh foods are generally more expensive than energy-dense items^(13,38)^. However, Roy *et al.* revealed that all foods available at this university's outlets had a high mark-up, regardless of nutritional quality^(8)^. Research shows that healthy food does not always have to be excessively price-reduced compared with unhealthy items^(13,39)^. If recipe changes to the ‘Budgie Meals’ cost retailers money for purchasing new ingredients, an increase in unhealthier items’ price (e.g. increasing the meal price by 10 and 20 % when choosing French fries^(5)^) could be implemented to offset any potential profit losses. Overall, the importance of price and a limited selection of healthy options for young adults are evident. Nutritional changes to the ‘Budgie Meal’ price-reduced meal initiative can ultimately improve access to healthy food for young adults at universities, and subsidies on healthier foods can significantly increase the purchase and consumption of promoted products^(32,40)^.

Previous studies have found that price reductions on low-fat snacks resulted in increased purchases from vending machines in university and other public settings^(28,29)^. Grech *et al.* found that healthy option sales increased when these items’ availability was increased in interventional studies^(41)^. The present study builds on French *et al.*'s work by using an observational model to establish more accurate responses to pricing strategies in a targeted population and evaluate the long-term sustainability of the price-reduced meals’ sales trajectories^(13,28,29,42)^. Small changes need to be made to have healthful food items available in the tertiary food environment while ensuring that the food remains tasty, cheap and convenient for young adults. The results show that minor recipe changes can be made to the ‘Budgie Meal’ items to improve their nutritional quantity while ensuring that taste is not compromised. Recipe changes include incorporating vegetables within dishes, using lite versions of ingredients and baking instead of frying. These recommended changes would not dramatically alter the taste, but the meal's healthfulness would be improved. Moreover, these nutritional improvements could make the ‘Budgie Meals’ more attractive for consumers to purchase, as staff and students have sought an increase in the availability of healthier and cheaper food options on campus^(43)^.

### Strengths

The greatest strength of the present study was the real-world setting used. Most studies investigating price initiatives and the availability of healthy food items were experimental or predictive simulation models. Therefore, the present study adds to the literature by examining a pre-existing price-reduced meal available at a large urban university and investigating how these cheaper menu items could be nutritionally improved. The present study has observed natural human behaviours and responses to a price-reduced meal initiative, which cannot be performed in experimental conditions. An additional strength of the present study was the present study design, as three methodologies were used to address the research questions. The use of three research approaches meant that a range of data were collected on food purchasing determinants, awareness of the ‘Budgie Meal’, ‘Budgie Meal’ sales volumes and their nutritional quality. The variety of research approaches provided different ‘Budgie Meals’ measures and, ultimately, a foundation for future improvements to this price-reduced meal initiative.

### Limitations

More females participated in the survey than males. This indicates selection bias, as the findings may be biased from a female student's perspective, based on their disproportionate participation. A further limitation was the large proportion of purchases made were beverages, and the subsequent potential missed opportunity for exposure to the ‘Budgie Meal’ price-reduced meal initiative. Food sales data were collected for all consumers, and therefore, young adults were not the specific target of the food sales outcomes. However, most of these outlets’ customers fell under the young adult age group (18–35 years). Only 5 % of the sample interviewed purchased the reduced-price meal, and any conclusions based on their responses are unrepresentative of the potential total population. The results of the present study cannot be generalised considering the lack of power, convenience sample used and because population characteristics and environment can differ greatly across countries and sometimes within a country. Precise comparisons between survey data and food sales data are difficult to make, as survey data collection only spanned a few weeks of one semester, whereas food sales data spanned the entire semester. Lastly, food sales data are only a measure of purchase behaviour change, and it is unknown whether food purchases directly impact staff and students’ dietary intakes.

## Implications for research and practice

Taste is the largest food purchasing determinant, and the price is the most significant barrier to healthy purchases on a university campus for young adults. A limited selection of healthy options was also a significant barrier to healthy food purchases for participants. Study findings provided evidence to support the ‘Budgie Meal’ as an attractive strategy for increasing the availability and accessibility to healthful foods in the university's food environment

Interventions combining pricing strategies with offering attractive and tasty food products may be more likely to succeed in improving students’ food choices^(5)^. In the future, recipe alterations to make the ‘Budgie Meals’ healthier should be considered by retailers while ensuring that the taste remains appealing. An important consideration for future research is an investigation into the feasibility of retailers implementing the recommended changes to their menu items, in terms of revenue^(44)^. The feasibility of working with food retailers, dietitians and customers using a co-design study and testing the acceptability of recipe alterations could be examined in the future^(45)^. This would examine whether the university food environment can alleviate the barriers of cost and lack of healthy options for young adults, and small changes such as the incorporation of vegetables may not have a significant impact on taste quality. Survey findings also revealed that the awareness of the ‘Budgie Meal’ initiative at the POP and the influence of the price-reduced meal initiative on purchases were poor, indicating that increased promotion at the POP is needed. In future research, improved promotion of the ‘Budgie Meal’ at the POP could be investigated. Increased promotion is also required to address the declining sales trend. The fidelity of implementation to ensure that ‘Budgie Meal’ programme promotion materials are being used as planned (approx. weekly) and the sustainability of ‘Budgie Meals’ through interviews with participating vendors can be evaluated in future research. Furthermore, suggested nutritional improvements may benefit attracting new customers seeking affordable, healthy food items on campus.

While the utilisation of the ‘Budgie Meal’ is a promising strategy to improve the quality of incentivized food available within the university, further research is required to assess the viability of implementing such nutritional improvements across food outlets. Specifically, collaboration with retailers and customers is needed to establish the economic feasibility, any potential revenue losses and testing taste acceptability of recipe alterations. With more evidence, the university food environment can be improved upon to ensure that healthful food are readily available, accessible and incentivized for university staff and students.
